# Cigarette smoke sustains immunosuppressive microenvironment inducing M2 macrophage polarization and viability in lung cancer settings

**DOI:** 10.1371/journal.pone.0303875

**Published:** 2024-05-22

**Authors:** Francesca Bianchi, Valentino Le Noci, Giancarla Bernardo, Nicoletta Gagliano, Graziano Colombo, Michele Sommariva, Michele Palazzo, Isabella Dalle-Donne, Aldo Milzani, Serenella Pupa, Elda Tagliabue, Lucia Sfondrini

**Affiliations:** 1 Department of Biomedical Sciences for Health, University of Milan, Milan, Italy; 2 U.O. Laboratorio di Morfologia Umana Applicata, IRCCS San Donato, Milan, Italy; 3 Department of Biosciences, University of Milan, Milan, Italy; 4 Unit of Microenvironment and Biomarkers of Solid Tumors, Department of Experimental Oncology, Fondazione IRCCS Istituto Nazionale dei Tumori, Milan, Italy; 5 Department of Medical Oncology, Fondazione IRCCS Istituto Nazionale dei Tumori, Milan, Italy; UNIVERSITY OF CENTRAL FLORIDA, UNITED STATES

## Abstract

**Background:**

It is amply demonstrated that cigarette smoke (CS) has a high impact on lung tumor progression worsening lung cancer patient prognosis and response to therapies. Alteration of immune cell types and functions in smokers’ lungs have been strictly related with smoke detrimental effects. However, the role of CS in dictating an inflammatory or immunosuppressive lung microenvironment still needs to be elucidated. Here, we investigated the effect of *in vitro* exposure to cigarette smoke extract (CSE) focusing on macrophages.

**Methods:**

Immortalized murine macrophages RAW 264.7 cells were cultured in the presence of CS extract and their polarization has been assessed by Real-time PCR and cytofluorimetric analysis, viability has been assessed by SRB assay and 3D-cultures and activation by exposure to Poly(I:C). Moreover, interaction with Lewis lung carcinoma (LLC1) murine cell models in the presence of CS extract were analyzed by confocal microscopy.

**Results:**

Obtained results indicate that CS induces macrophages polarization towards the M2 phenotype and M2-phenotype macrophages are resistant to the CS toxic activity. Moreover, CS impairs TLR3-mediated M2-M1 phenotype shift thus contributing to the M2 enrichment in lung smokers.

**Conclusions:**

These findings indicate that, in lung cancer microenvironment of smokers, CS can contribute to the M2-phenotype macrophages prevalence by different mechanisms, ultimately, driving an anti-inflammatory, likely immunosuppressive, microenvironment in lung cancer smokers.

## Background

Lung cancer is one of the most common cancers in the world and several research efforts are ongoing to improve patients’ clinical outcome. Tobacco is the leading carcinogen for lung cancer and, although smoking rates have decreased across the world, nearly 90% of the world’s lung cancers are due to cigarette smoking. Moreover, despite decades of progress in reducing cigarette consumption, it is estimated that nearly 1 billion smokers are still present, especially in low- or middle-income countries [1].

All histologic subtypes of lung cancer have been associated with smoking. Particularly, smokers account for 80% of patients with Non-Small-Cell Lung Cancer (NSCLC) [2, 3], the most frequent subtype of lung cancer [4]. The carcinogenic role of smoke has been extensively studied, and several mechanisms by which it induces neoplastic transformation are well known [5]. Moreover, cigarette smoke (CS) also plays a significant role in lung tumor progression and it has been well attested that smokers with lung cancer have a worse prognosis compared to non-smoking patients [6–8]. Besides the occurrence of comorbidities in smokers and the higher frequency of mutations in actionable driver genes [9], several other mechanisms have been to date described to explain the effect of CS on tumor growth. Among these, a role of altered functions of immune cells in smokers’ lungs has also been considered and this topic represents an emerging aspect in the lung cancer research. Indeed, besides having different molecular profiles, lung cancers in smokers and never-smokers show different composition of immune cells in the tumor microenvironment (TME) [10], which has been related to clinical outcomes [11] and propose to likely impacts on the susceptibility to novel therapeutic treatments, particularly immunotherapy.

Collectively, the effect of smoking on the immune lung microenvironment is related to the recruitment of inflammatory cells (neutrophils, macrophages, eosinophils) and the reduction of cell populations of other subtypes (Natural Killer cells, Dendritic cells, B cells) leading to an immunosuppressive state [11, 12].

Alveolar macrophages (AMs) constitute the dominant immune cells in lungs, primarily responsible for the lung immune defense. These cells are key players in the balance between defense against pathogens and tolerance toward innocuous stimuli [13]. In the tumor microenvironment of various cancers M1 “classically activated” macrophages, which have pro-inflammatory and anti-tumoral functions, and M2 “alternatively activated” macrophages, which have anti-inflammatory and pro-tumoral functions coexist because of a continuous process of naïve M0 macrophage polarization and a high plasticity of M1 and M2 phenotypes [14]. However, many studies widely demonstrated the prevalence of M2 polarized cells in tumor-associated macrophages (TAMs) [15, 16] and, accordingly, in NSCLC about 70% of TAMs are M2-like [17, 18].

Several TLR ligands have been shown to have an anti-tumor effect in different types of cancer and currently represents an effective therapeutic strategy to boost the anti-tumor innate response [19–22]. Particularly, the administration of TLR3 ligand has been shown to revert the M2-macrophages to M1-phenotype and regressed the tumor growth in murine models [23, 24]. However, we recently observed that TLR3 expression on immune cells, the majority of which resulted to be macrophages, is associated with poor overall survival in a cohort of 194 patients with early-stage NSCLC [25]. Therefore, whether the activation of TLR3 can promote anti-tumor M1-phenotype macrophages, still remains an open question particularly in smoking context.

In the present study, we investigated the effect of *in vitro* exposition to cigarette smoke extract (CSE) on macrophages polarization, features, activation by TLR3 ligand and on their interaction with tumor cells in immortalized murine macrophages RAW 264.7 and Lewis lung carcinoma (LLC1) murine cell models.

## Methods

### Cells and cell culture

Immortalized murine macrophages (RAW 264.7) and Lewis Lung Carcinoma (LLC) cell lines were routinely maintained at 37°C in 5% CO_2_ atmosphere in Dulbecco’s Modified Eagle Medium (DMEM) (Gibco) supplemented with 10% fetal bovine serum (FBS) (Gibco). Poly(I:C) was added to the culture medium at 100 ug/ml final concentration. To determine cells proliferation, Sulforhodamine B (SRB) assay was used, as we previously described [26, 27]. Absorbance at 510 nm was measured and expressed as optical density (OD). Viability of cells in the presence or not of CSE in culture medium has been evaluated by counting the number of viable cells by Trypan Blue staining after harvesting from 6-well plates; 5 x 10^5^ cells/well initial quantity of cells seeded. To obtain 3D-spheroids, 5 x 10^4^ cells were seeded in 24-well plates or 1 x 10^4^ total cells (1 x 10^4^ RAW cells or 7 x 10^3^ RAW cells + 3 x 10^3^ LLC1 cells respectively), were seeded in 96-well plates, (Costar, Corning Incorporated) coated with 1% agarose in DMEM culture medium [28, 29]. Samples were imaged under a Nikon ECLIPSE Ti-TimeLapse microscope (Nikon); data were analysed using Adobe Photoshop 7.0 by measuring the major diameter of each spheroid using the "ruler" tool. The ratio was calculated between the diameters of the spheroids in the presence of CSE compared to those of the spheroids cultured without CSE in the culture medium.

### Preparation of whole-phase cigarette smoke extract (CSE)

Whole-phase CSE from Kentucky 3RF4 reference cigarettes was prepared as previously described [30]. Mainstream smoke from one cigarette (10 puffs) was allowed to dissolve (for 10 s each puff) in 10 ml of 50 mM potassium phosphate buffer (PBS), pH 7.4. The resultant dark yellow solution was defined as 100% whole-phase CSE and was filtered through a 0.22-μm Millipore filter (Bedford, MA) to remove bacteria and large particles. The pH of the whole-phase CSE was adjusted to 7.4 by addition of 2 M sodium hydroxide solution. To ensure standardization between experiments and batches of CSE, CSE preparations were made uniform by measurement of absorbance at 340 nm. CSE was freshly prepared immediately before use for each experiment and diluted to an appropriate concentration with 50 mM PBS.

### Reverse transcription and real-time PCR

Gene expression analysis assessments were conducted by extracting RNA from cells cultured under different conditions. Initial quantity of 5*10^5^ cells/well in 6-well plates. RNA from RAW 264.7 cells was isolated using QIAzol (Qiagen) following the manufacturer’s instructions, as we previously described [31, 32]. The concentration of RNA was evaluated by the spectrophotometer NanoDrop 2000 (Thermo Fisher Scientific). Reverse transcription was performed using the High-Capacity RNA-to-cDNA Kit and real-time PCR was performed using TaqMan Fast Universal PCR Master Mix and SDS 2.4 on a 7900HT Fast Real-Time PCR System (all by Applied Biosystems-Thermo Fisher Scientific), with the following TaqMan gene expression assays (Applied Biosystems-Thermo Fisher Scientific): *Stat1* (Mm00439531_m1); *Stat6* (Mm01160477_m1); *IFN-β1* (Mm00439552_s1); *IL-10* (Mm01288386_m1); *Irf4* (Mm00516431_m1); *Irf5* (Mm00496477_m1); *TLR3* (Mm01207404_m1); Arginase-1 (Mm47588_m1); Nos2 (Mm00440502_m1). Gene expression was normalized to *B2m* (Mm00437762_m1). PCR data were analyzed using the 2^-ΔCt^ method.

### Microscopy analysis

Cells were stained with carboxyfluorescein succinimidyl ester (CFSE) (Life Technologies) following manufacturer’s instructions. Briefly, the cells were stained with PKH26 (Sigma-Aldrich) following manufacturer’s instructions. The samples were imaged under a Leica TCS SP8 X confocal laser scanning microscope (Leica Microsystems GmbH) and the data were analysed using Leica LAS X rel. 3.1.1 (Leica Microsystems GmbH), or were imaged under Nikon Eclipse TE 2000-S and analysed using the NIS Elements analysis software system (Nikon).

### Flow cytometry

Cytofluorimetric assessments were conducted from RAW cells cultured under different conditions. This was done in 6-well plates (Costar, Corning Incorporated), seeding an initial quantity of 5 x 10^5^ cells/well. RAW cells were harvested and cell suspensions were washed with PBS 1X FBS 2% and then labeled with Live/Dead (1:1000) (Live/Dead Fixable Near-IR Dead Cell Stain Kit, Invitrogen by Thermo Fisher Scientific) for 30 minutes at 4°C in the dark. Cells were washed with PBS 1X FBS 2% and stained with the following antibodies: anti-mouse CD80 APC (MACS Miltenyi Biotech); anti-mouse CD86 PE (BD Biosciences Pharmingen). After washing, cells were fixed with 1% formalin 15 minutes at room temperature. Cells were examined using a FACSCanto flow cytometer (BD) and data were analyzed using FlowJo software (TreeStar). Plotted ratio was calculated between the percentage value of RAW cells with positive M2 phenotype for the expression of CD80 and CD86, respectively (see gating strategy described in S1 Fig in S1 File), divided by the percentage value of untreated RAW cells with positive M2 phenotype without Poly(I:C) and/or CSE treatment.

### Statistical analysis

Differences between groups were determined by two-tailed student’s t-test. A two-sided p-value of <0.05 was considered to be significant. The statistical analysis was performed using the GraphPad Prism 5.01 package (GraphPad).

## Results

### Analysis of cigarette smoke effect on macrophage polarization

**Fig 1:**
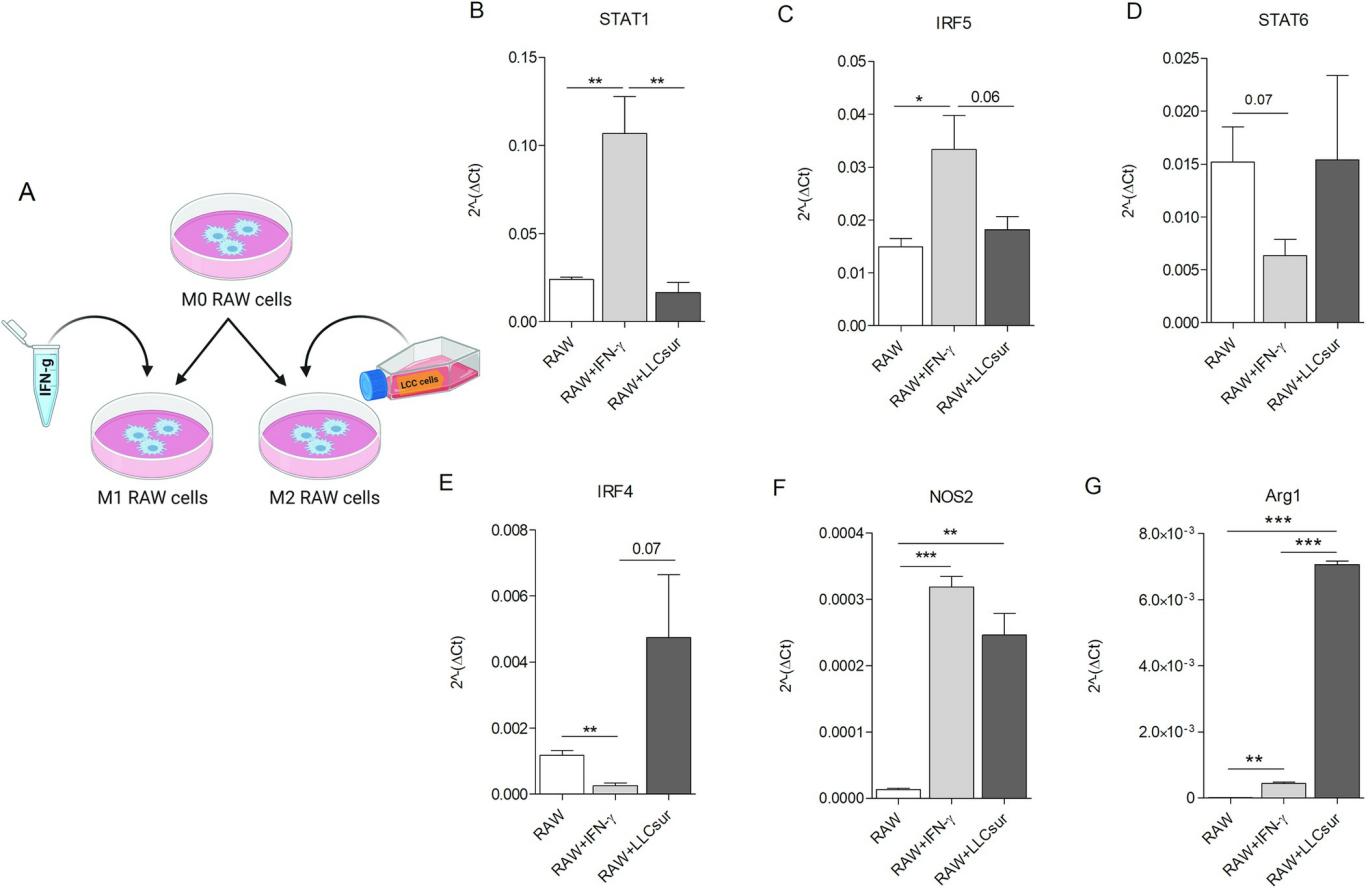
Expression levels of transcription factors driving M1, M2 phenotypes in macrophages. RAW 264.7 were seeded and then left untreated (M0) or conditioned with IFN-γ (5 ng/mL) (M1) or with 5-days metabolized LLC supernatant (M2) (A), created with BioRender.com. Real-Time PCR analysis of transcription factors STAT1 (B), IRF5 (C), STAT6 (D), IRF4 (E), NOS2 (F) and Arginase-1 (Arg1) (G) (min. 3 independent experiments. Mean ± SEM, Unpaired t-test, p-value * <0.05, ** <0.01).

Untreated RAW 264.7 mouse macrophage (RAW) cell line, considered as M0 macrophages, were stimulated with IFN-γ (5 ng/mL) or 5-day metabolized LLC supernatant to induce M1 and M2 phenotype, respectively (Fig 1A).

Real-Time PCR analysis revealed higher mRNA levels of Stat1, Irf5 and Nos2, M1 macrophage markers [33], in IFN-γ-polarized RAW cells compared to RAW cells conditioned with LLC supernatant (Fig 1B, 1C and 1F). Conversely, considering well known M2 macrophage markers [33], significant increased mRNA levels of Arg1, and increased mRNA levels of STAT6 and Irf4 albeit not statistically significant, were observed in LLC supernatant-treated RAW cells, compared to those treated with IFNγ (Fig 1D, 1E and 1G). No evident morphological variations were noticed among the different experimental groups (S2 Fig in S1 File).

**Fig 2:**
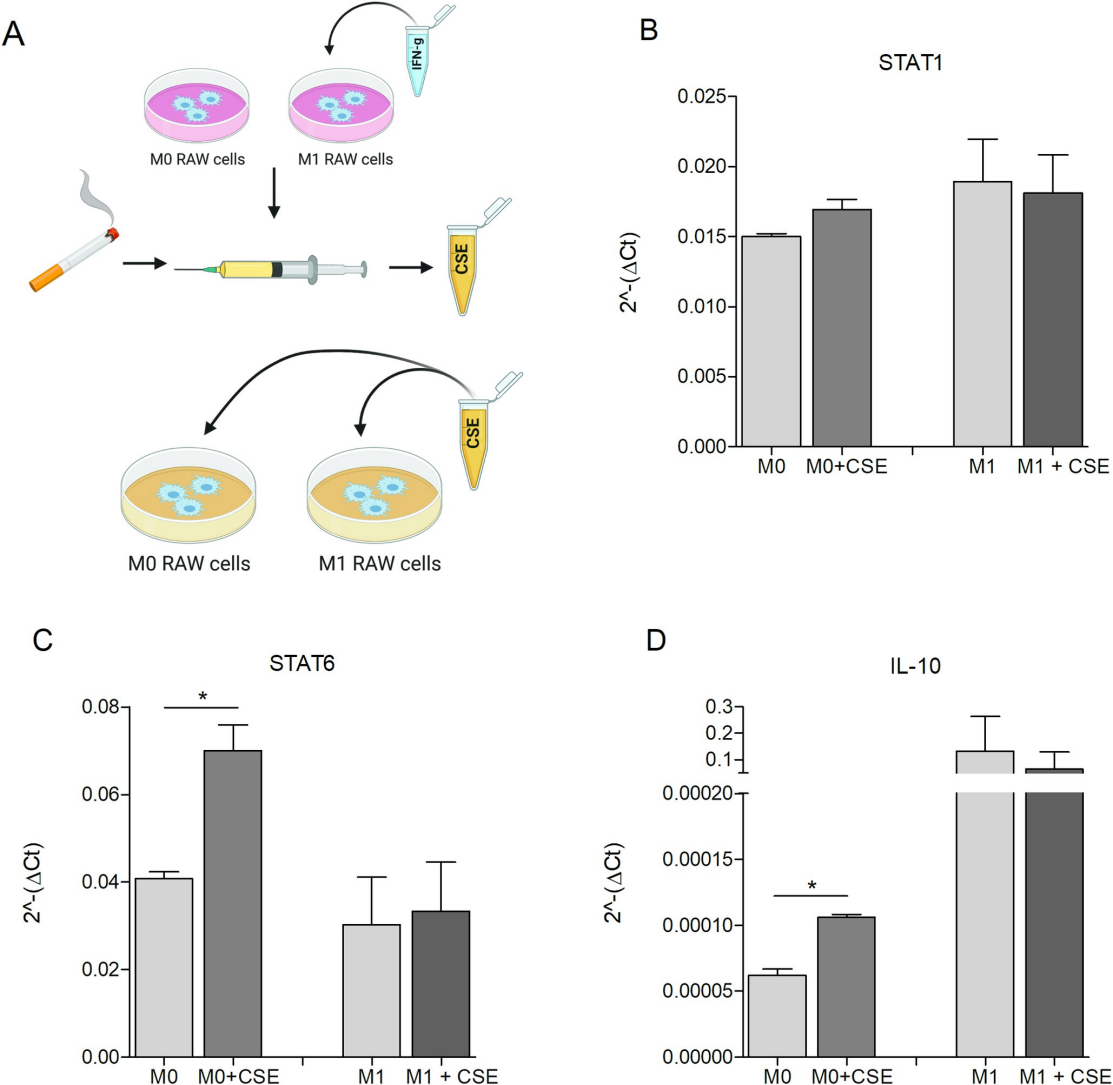
Analysis of phenotype shifting in macrophages in vitro exposed to CSE. RAW 264.7 were seeded and left untreated (M0) or conditioned with IFN-γ (5 ng/mL) (M1) and treated with CSE 5% for 48 hours (A), created with BioRender.com. Real-Time PCR analysis of STAT1 (B), STAT6 (C) and IL-10 (D) (min. 2 independent experiments. Mean ± SEM, Unpaired t-test, p-value * <0.05, ** <0.01).

In order to investigate whether CS can impact on M1/M2 macrophage polarization or can promote the switch from M1 to M2 phenotype, M0 and M1-polarized RAW cells were treated with CSE 5% for 48 hours (Fig 2A). No changes in Stat1 mRNA were observed either in M0 or in M1 RAW cells treated with CSE (Fig 2B). A higher expression of Stat6 was observed in M0 cells treated with CSE compared to the untreated counterpart, whereas no significant modulations in Stat6 expression was detected in M1 cells treated with CSE compared to control (Fig 2C).

Since the increased expression of Stat6 suggests a possible shift towards an immunosuppressive M2 phenotype, we then evaluated the expression of IL-10, another M2 marker [34]. As shown in Fig 2D, an increased expression of IL-10 was observed in M0 cells treated with CSE, but not in M1 cells. Moreover, CD86 and CD80 activation markers were not affected by CSE treatment (S3 Fig in S1 File).

These results suggest that CSE promotes a shift from M0 to M2 phenotype, whereas does not induce a reprogramming of M1 to M2 macrophages.

### Analysis of cigarette smoke effect on M2-M1 phenotype shift mediated by TLR3 activation

**Fig 3:**
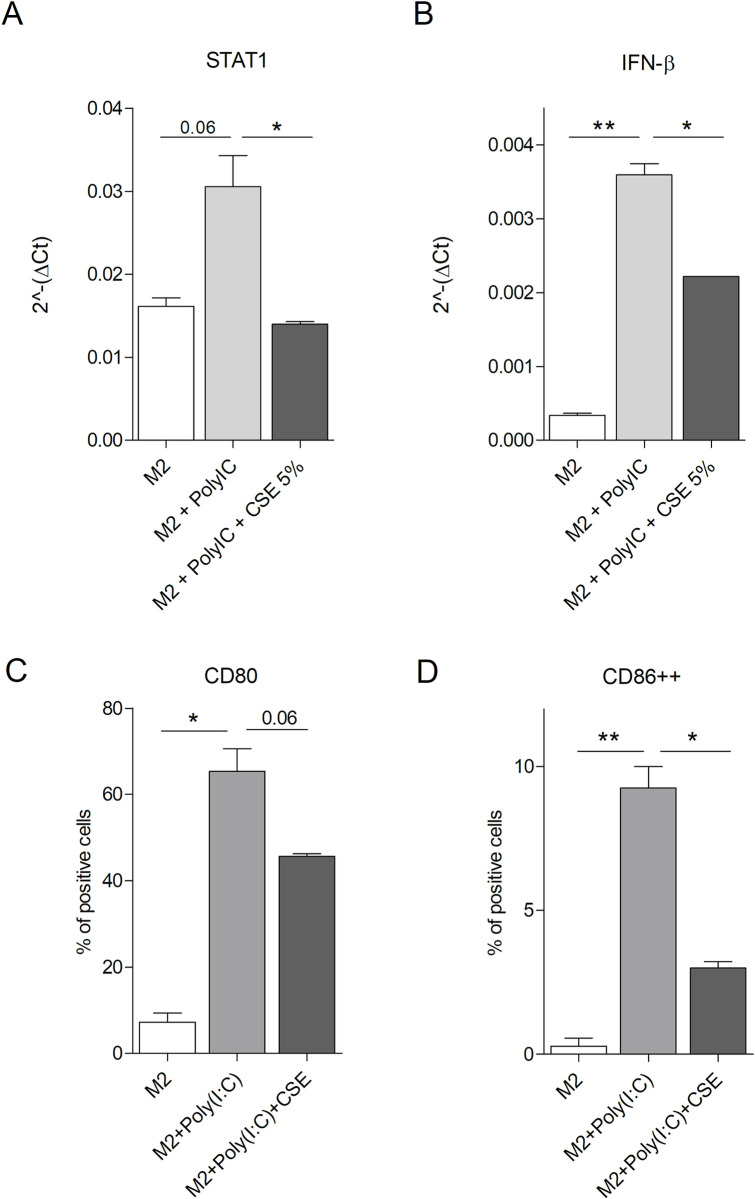
Analysis of phenotype shifting in M2-phenotype macrophages in vitro stimulated with TLR3 ligand and exposed to CSE. RAW 264.7 were seeded, conditioned with metabolized LLC supernatant (M2), stimulated or not with Poly(I:C) (100 ug/ml) and treated or not with CSE 5%. After 48 hrs, the expression level of STAT1 (A) and IFN-β (B) have been analyzed by Real-Time PCR (Representative of 5 and 2 independent experiments. Mean ± SEM, Unpaired t-test, p-value * <0.05, ** <0.01). After 24 hrs, the percentage of cells positive for CD80 (C) and CD86 (D) maturation markers has been analyzed by flow-cytometry (2 independent experiments. Mean ± SEM, Unpaired t-test, p-value * <0.05, ** <0.01). Plotted values are the percentage values of RAW cells with positive M2 phenotype for the expression of CD80 and CD86, respectively, with Poly(I:C) and/or CSE treatment.

To assess the effect of CSE on TLR3 agonist capability to revert M2 macrophages to M1 phenotype, M2-polarized RAW cells were treated with Poly(I:C), a synthetic TLR3 agonist, in the presence or not of CSE 5% for 48 hours. Higher levels of Stat1 mRNA were observed in M2 cells treated with Poly(I:C) compared to untreated controls (Fig 3A), indicating a shift towards M1 phenotype. A lower level of Stat1 was observed in M2 cells treated with Poly(I:C) and CSE compared to M2 cells treated with Poly(I:C) alone, suggesting that CSE can counteract the shift from M2 to M1 phenotype mediated by TLR3 triggering (Fig 3A). To confirm this observation, the expression of IFN-β1 was investigated in M2-polarized RAW cells treated or not with Poly(I:C) and CSE. Poly(I:C) administration strongly induced IFN-β1 expression, whereas CSE treatment hijacked the capability of the TLR3 agonist to skew M2 macrophages to M1 (Fig 3B).

Flow cytometry analysis showed a significantly higher expression level of CD80 and CD86 in M2-polarized RAW cells treated with Poly(I:C) compared to untreated control (Fig 3C and 3D) confirming the acquisition of a M1 phenotype. A decrease of CD80 expression in M2 cells treated with Poly(I:C) and CSE was observed compared to M2 cells treated with Poly(I:C) alone. Moreover, although Poly(I:C) increased CD86 expression in all macrophage population, it was possible to observe a reduction in CD86++ fraction in Poly(I:C)/CSE combination group. These data suggest the CSE can impair M2-M1 phenotype shift mediated by TLR3 agonist.

### Analysis of cigarette smoke effects on macrophage features

**Fig 4:**
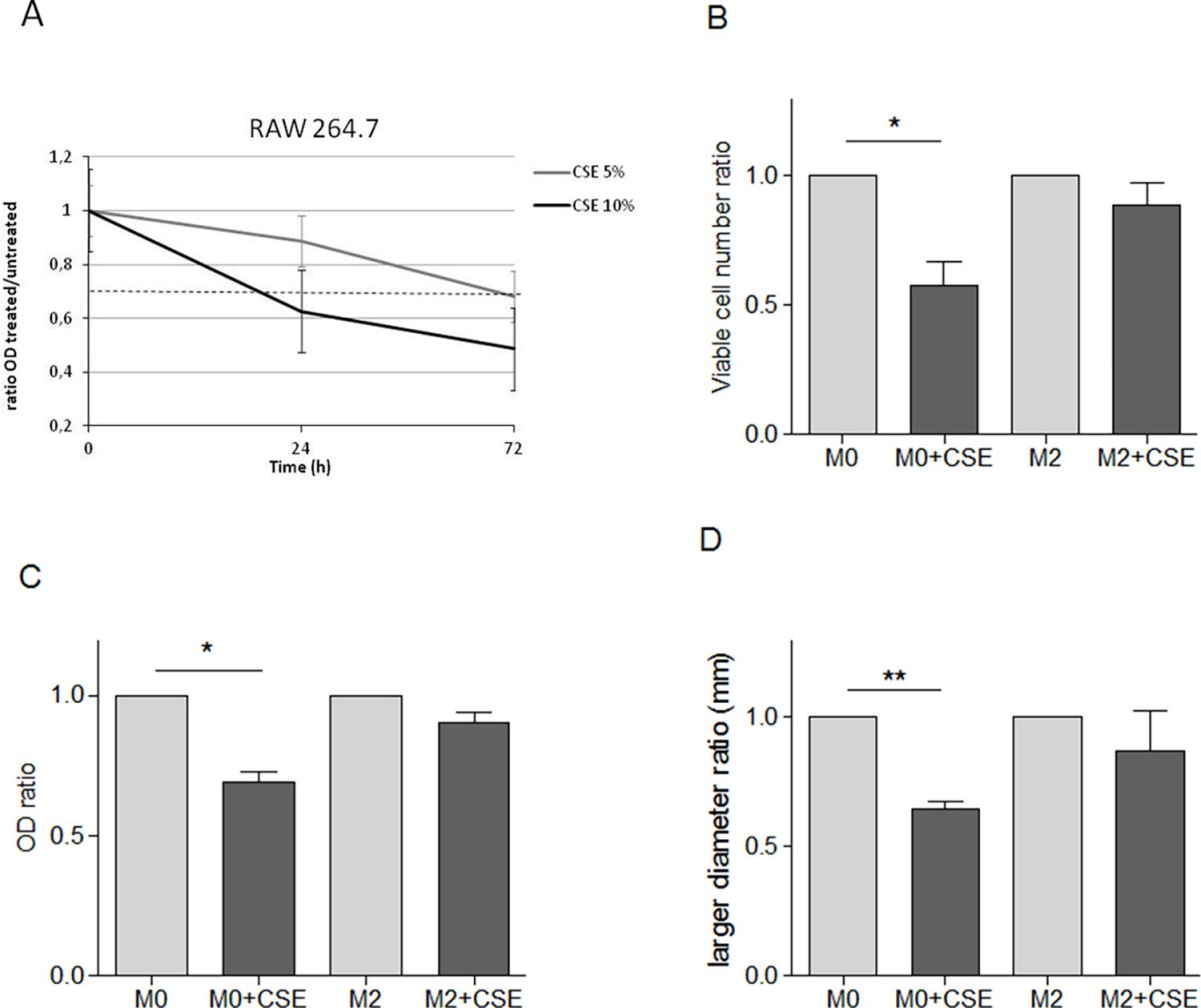
Analysis of viability and 2D and 3D cells growth capability of M0/M2-phenotype macrophages in vitro exposed to CSE. RAW 264.7 were seeded and treated or not with CSE 5%-10% for different time. Growth capability of CSE-treated vs. untreated macrophages has been evaluated by SRB assay (A). RAW 264.7 were seeded, conditioned (M2) or not (M0) with metabolized LLC supernatant and treated or not with CSE 5%. Viability of M0- and M2-phenotype macrophages in the presence or not of CSE in culture medium has been evaluated by counting the number of viable cells by Trypan Blue staining (B) and by SRB assay (C). M0- and M2-phenotype macrophages were seeded in plates coated with 1% DMEM agarose, to form 3D-spheroids as described in Materials and Methods, and immediately treated with CSE 5% for 48 hrs. Images have been acquired and the size of formed 3D-spheroids has been evaluated (D). Specifically, data were analysed using Adobe Photoshop 7.0 by measuring the major diameter of each spheroid using the "ruler" tool. The ratio was calculated between the diameters of the spheroids in the presence of CSE compared to those of the spheroids cultured without CSE in the culture medium. (min. 3 independent experiments. Mean ± SEM, Unpaired t-test, p-value * <0.05, ** <0.01).

To investigate the effects of CS on macrophages features, viability of the RAW cells has been assessed in the presence or not of CSE in culture medium. CSE impacts RAW cells growth in dose- and time-dependent manner (Fig 4A). A significant strong reduction in the number of viable M0 cells was observed upon CSE treatment compared to untreated cells. Interestingly, no significant reduction of the number of M2 cells treated with CSE was observed (Fig 4B). The sensitivity of the RAW M0 phenotype, and the resistance of the RAW M2 phenotype to the CSE were confirmed by SRB growth assay (Fig 4C).

Spheroids were settled as a 3D-model to study the effect of CS on macrophages plasticity (see Materials and Methods). Compared with traditional 2D cell culture, 3D spheroids can closely mimic the architecture and physiology of *in vivo* cell-cell interaction as an *in vitro* model and are a widely used tool for studying the response of tissues to external factors, such as smoke diffusion and interaction with cells in non-adherent conditions.

To evaluate whether smoke impairs the formation process of RAW 3D-spheroids, M0 or M2 RAW cells were seeded in plates coated with 1% agarose and immediately treated with CSE 5%. In the presence of CSE, M0 spheroid resulted significantly smaller than the untreated counterpart. In contrast, the ability of M2 RAW to assembly 3D-spheroids was not affected by CSE (Fig 4D), suggesting a lower sensibility to CS of macrophages conditioned by tumor cells.

However, our experiments revealed that CSE 5% added to culture medium once the 3D-spheroids is established did not influence the diameter of already established RAW 3D-spheroids (S4 Fig in S1 File), possibly to the lower ability of smoke to penetrate in already established 3D-structures.

### Analysis of macrophage-tumor cells interaction following exposure to cigarette smoke extract

**Fig 5:**
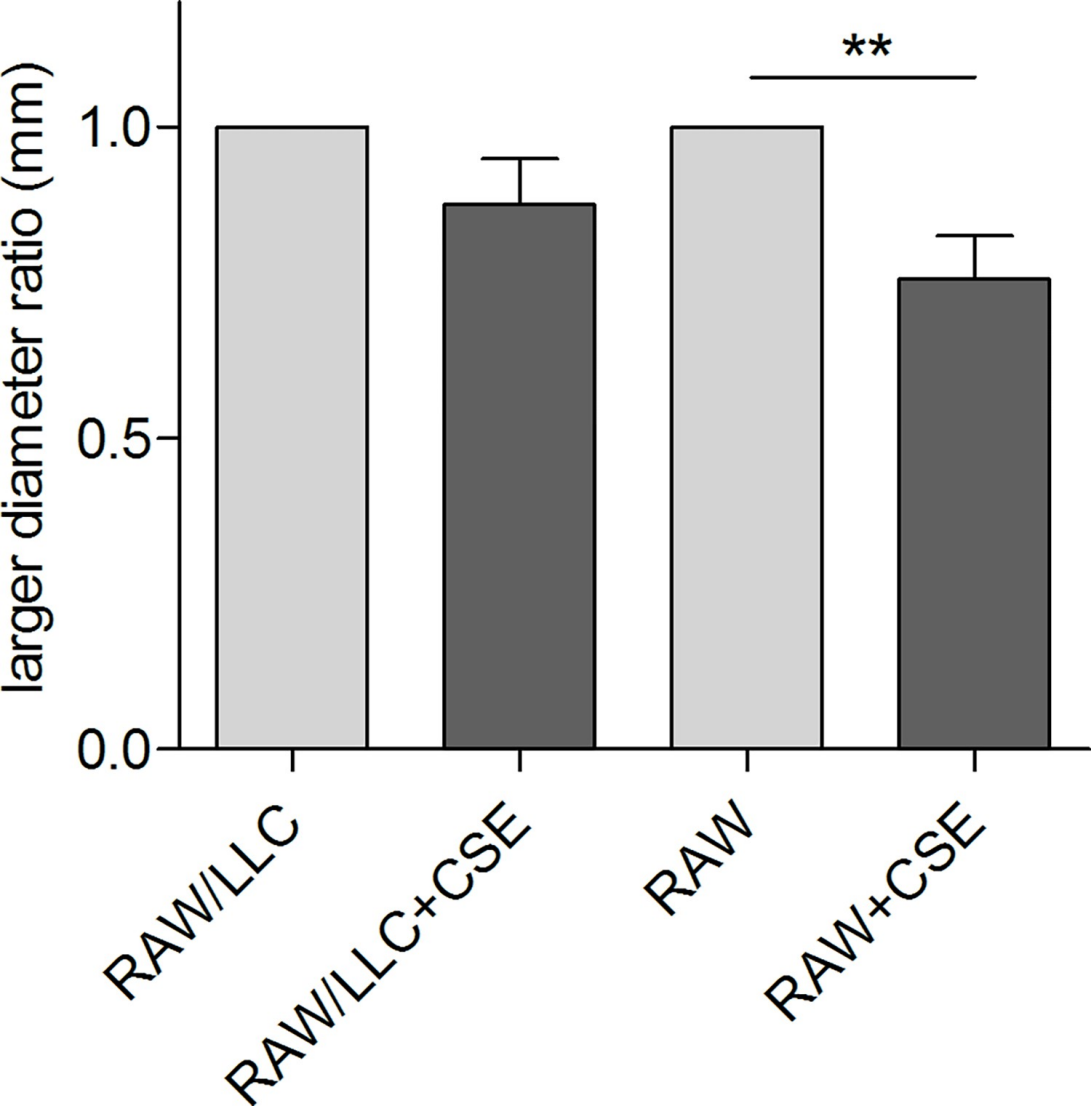
Analysis of 2D and 3D cells growth capability of M0/M2-phenotype macrophages in vitro co-cultured with LLC cells. RAW 264.7 and LLC cells were seeded together in plates coated with 1% DMEM agarose, to form 3D-spheroids as described in Materials and Methods, and immediately treated with CSE 5% for 48 hrs. Images have been acquired and the size of formed 3D-spheroids has been evaluated. Specifically, data were analysed using Adobe Photoshop 7.0 by measuring the major diameter of each spheroid using the "ruler" tool. The ratio was calculated between the diameters of the spheroids in the presence of CSE compared to the mean of those cultured without CSE in the culture medium (A). 2D-growth capability of RAW 264.7 cells in vitro co-cultured with LLC cells, in the presence or not of CSE in culture medium has been evaluated by SRB assay (B). (Mean ± SEM, Unpaired t-test, p-value * <0.05, ** <0.01).

To investigate macrophages and tumor cells interaction in the presence of CS, RAW and LLC cells were seeded together in plates coated with 1% agarose and immediately treated with CSE 5%. CSE slightly and not significantly reduced the size of 3D-spheroids containing both RAW and LLC cells (Fig 5). Notably, a significantly higher impairment of 3D-spheroid formation caused by CSE was observed in RAW cultured alone than in RAW co-cultured with LLC (25% of reduction vs. 13%, respectively).

**Fig 6:**
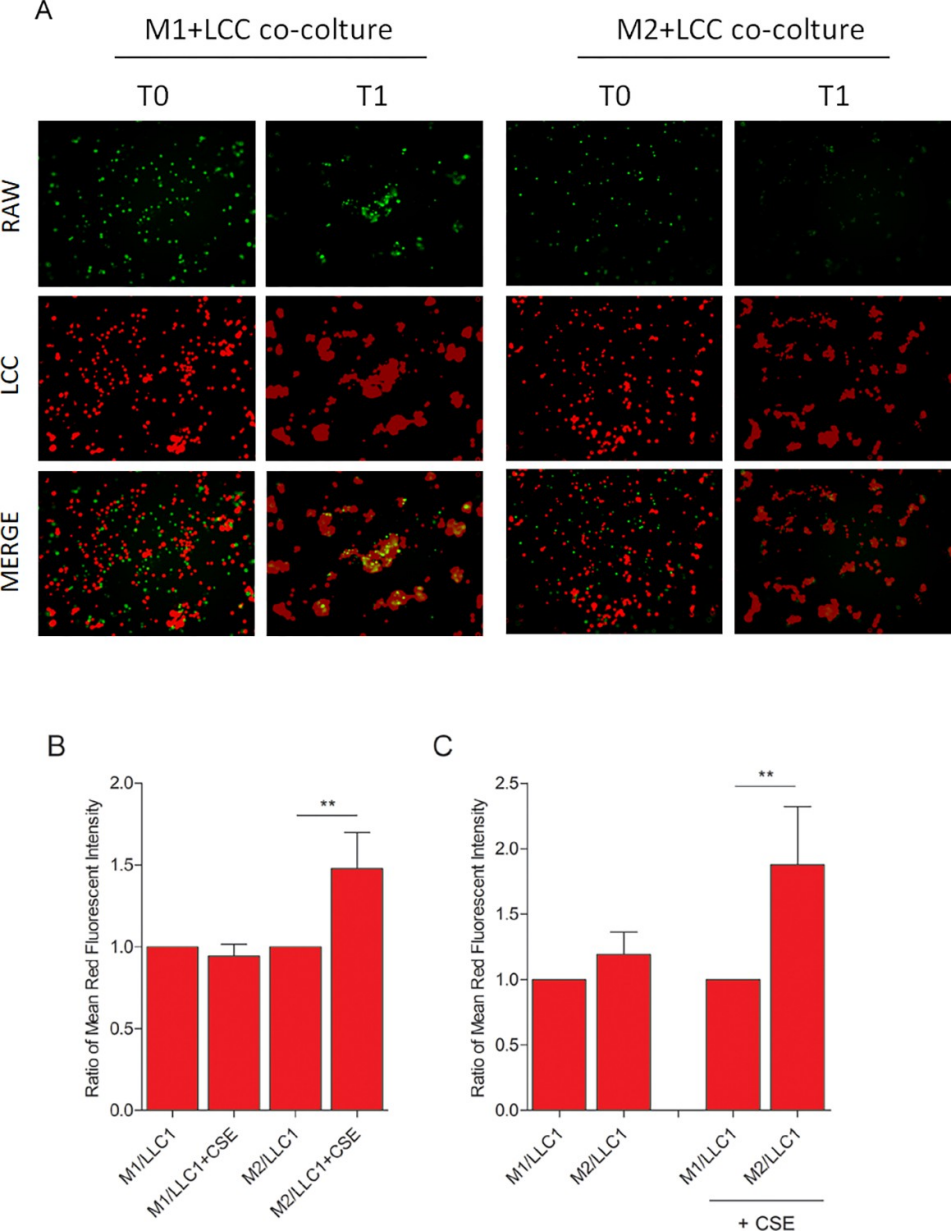
Analysis of M1/M2-phenotype macrophages interaction with LLC cells in in vitro 2D co-culture. M1- or M2-phenotype RAW 264.7 (green stained) and LLC cells (red stained) were seeded together, and images have been acquired by confocal microscopy at the beginning of co-culture (T0) and after 24 hours of co-culture in the presence of CSE 5% (T1) (A). Quantitative analysis by fluorescent microscopy of red fluorescence intensity in the M1/LLC and M2/LLC cells co-cultures exposed or not to CSE (B, C). Images were analysed by using the multi-channel binary software analysis toolbox for 2D measurement of intensity (Mean ± SEM, Unpaired t-test, p-value * <0.05, ** <0.01; 3 independent experiments). In Fig 6B, the plotted ratio was calculated as follows: for each of the three independent experiments, the mean signal intensity of red fluorescence, corresponding to LLC cell labeling, acquired for each well in co-culture with RAW M1 or RAW M2, was calculated. Then, for each of the three experiments, the ratio was calculated between the mean calculated in the samples in the presence of CSE versus those without CSE. Finally, the mean and standard error of the calculated ratios were plotted. Similarly, in Fig 6C, the plotted ratio was calculated as follows: for each of the three independent experiments, the mean signal intensity of red fluorescence, corresponding to LLC cell labeling, acquired for each well in co-culture with RAW M1 or RAW M2, was calculated. Then, for each of the three experiments, the ratio was calculated between the mean calculated in the samples co-culture with RAW M2 versus those with RAW M1, in presence or not of CSE. Finally, the mean and standard error of the calculated ratios were plotted.

To investigate macrophage-tumor cells interaction in the presence of CS, M1- and M2-induced RAW cells were co-cultured with LLC cells in the presence of CSE 5%. Before seeding, RAW cells were stained with a green fluorochrome (CFSE), while LLC cells were stained with a red fluorochrome (PKH26). Images were digitalized by confocal microscopy at the beginning of co-culture (T0) and after 24 hours of co-culture in the presence of CSE (T1) (Fig 6A). The analysis of the red-fluorescent signals in fluorescence images of the co-cultures exposed to the CSE showed a greater growth rate of the LLC1 in the presence of RAW M2 instead of M1, considering as a control both the LLC1 grown in the presence of RAW M2 without CSE (Fig 6B), and the LLC1 grown in the presence of RAW M1 with CSE (Fig 6C).

## Discussion

Aim of this work was to investigate the effects of CSE exposure on macrophages polarization, features and viability. We observed that CSE exposure can polarize M0 macrophages towards a M2-phenotype.

Cigarette smoke consists of tar and gas phase: the latter is toxicologically important because it can pass through lung alveolar epithelium to enter the circulation. Whole-phase cigarette smoke extract (CSE) is a model system widely used for studying *in vitro* effects of CS [35, 36] whose administration invokes an inflammatory response in the lung similar to that observed after CS exposure [37].

To explore the effects of CSE on M1 and M2 macrophage phenotype, RAW 264.7 mouse macrophage cell line was treated with IFN-γ to obtain M1 phenotype [38], or metabolized LLC supernatant to induce M2 phenotype [39, 40]. The use of tumor conditioned medium to stimulate macrophages is a well-recognized strategy to shape macrophages towards a phenotype closely resembling that found in the tumor microenvironment [41]. The effect of IFN-γ in inducing a shift in macrophage phenotype towards a pro-inflammatory M1 direction has been extensively demonstrated [42–45]. The main effectors of IFN-γ signaling are STAT1 and IRF5, transcription factors which act upstream of the regulation of the expression of genes such as iNOS [46–48].

In our experimental model, we observed that CSE is not able to shift macrophages from M1- to M2-phenotype. However, in lung cancer microenvironment several other factors, such as time and concentration of CSE exposure, affect M1-M2 phenotype balance and may interfere with CS capability to induce M1-M2 macrophages phenotype shift.

The effect of CS on macrophage polarization has attracted considerable attention but is still controversial, considering the reported opposite ability of CS exposure to promote the acquisition of an anti-inflammatory as well as a pro-inflammatory phenotype in macrophages. Following CSE exposure, NF-κB and JAK2/STAT3 pathways have been described to decrease the levels of inflammatory mediators, such as reactive oxygen species (ROS) and nitric oxide (NO), paralleled by the reduction of the expression of M1-related cytokines, as TNF-α, IL-12p40, and the increase of the M2 cytokines IL-10, IL-6 and TGF-β [49, 50]. Conversely, it was reported that CSE and nicotine treatments could enhance the expression of pro-inflammatory cytokine IL-8 both in human and murine macrophages [51, 52]. More recently, Feng and colleagues showed that polarization towards inflammatory M1 phenotype is initially induced by smoke and that then the expression of Arg-1 gradually increased indicating a progressive shift towards a M2 phenotype [53].

Similarly, conflicting conclusions also come from studies in human specimens. In AM from bronchoalveolar lavages of healthy smokers, a transcriptional profile of M1-deactivated and partially M2-polarized macrophages was observed [54]. However, in normal lungs most AM were non-polarized and the percentage of both M1 and M2 increased progressively with smoking. More interestingly, upon smoke exposure, AM can express simultaneously markers of M1 and M2 polarization [55].

These data can lead to the hypothesis that CS effect on macrophages polarization is a dynamic process and that, after prolonged exposure, a reprogramming of macrophages towards an M2-type phenotype rather than the M1-type can occur in smoking conditions. However, the co-existence of M1 and M2 macrophages, even upon CS stimulation, cannot be excluded.

Innate immune cells are crucial to mount and sustain a proper anti-tumor immune response and, indeed, many therapeutic strategies aimed at boosting innate immune cells anti-cancer activity have been explored [56–58], such as the use of Toll-like receptors (TLRs) agonists for the success of currently proposed immunotherapy agents in lung cancer, especially immune-check points inhibitors [59, 60]. Besides several strategies we and others explored to boost anti-tumor innate immune activity [61]. For instance, Ligands of innate immunity, such as the Toll-like receptors (TLRs) [62], play a crucial role in changing the profile of macrophages and molding the anti-tumor activity of innate immune populations [63–65]. Among these, the relevance of TLR3 expression on immune cells in dictating lung cancer progression has been demonstrated, indicating TLR3 as a prognostic marker for early NSCLC [25, 32, 66, 67].

Poly(I:C), a synthetic analog of viral double-stranded RNA (dsRNA), is a TLR3 agonist that induces efficient anticancer activity acting on macrophages, by promoting a switch from M2 to M1 phenotype, eventually reducing tumor growth [23, 24]. In this context, we and others have demonstrated that the delivery of a TLR3 agonist into the bronchoalveolar space reduces the presence of M2-associated arginase- and IL-10-positive AMs in tumor-bearing lungs [68, 69], possibly through an IFN-αβ dependent mechanism, as suggested [24, 70]. Several mechanisms have been suggested to explain the M1 polarization induced by Poly(I:C): the upregulation in the expression of costimulatory molecules (e.g. CD80, CD86, CD40); the inhibition of co-inhibitory receptors (Tim-3) and the induction of IL-6, IL-12, TNFα [24]. Polarization of M2 macrophages toward M1 phenotype is and IFN-γ can greatly potentiate the effect of Poly(I:C), resulting in strong tumoricidal activity.

Our data indicate that CS impairs TLR3-mediated M2 to M1 skewing, since both the reduction of expression of STAT1 and IFN-β and of the activation markers CD80 and CD86 have been observed in macrophages treated with the combination of CSE and Poly(I:C).

The acute inflammatory responses caused by smoking was reported to depend on TLRs, via increasing their expression and responsiveness [71–73]. However, this scenario is not always clear. Koarai and colleagues observed an increase of TLR3-positive AMs in smokers compared to non-smokers, and that CSE potentiated the expression of TLR3 augmenting the release of IL-8, in cells treated with TLR3 ligand [74]. Conversely, several evidences indicate that CS can also impair TLR3 function [71, 75, 76], and, accordingly, it has been reported that CS negatively affect bacterial phagocytosis by macrophages [49, 77, 78]. Moreover, AMs of smokers showed reduced protein expression of TLR3 compared to those of never-smokers and the percentage of TLR3-positive cells inversely correlated with active smoking habits [79]. Finally, we did not observe a significantly different TLR3 expression on immune cells infiltrating NSCLC [25].

Consistently with the opposite effects of CS on the inflammatory status of tumor microenvironment, TLR3 activation induced by dsRNA that is released by CS-damaged cells can boost the inflammatory microenvironment, which could promote tumor growth. Conversely, TLR3 signaling also upregulates proinflammatory and anti-inflammatory cytokines, which support the immune tolerogenic status of the tumor.

In small airway epithelial cells, smoke exposure, by impairing TLR3 cleavage, strongly inhibited the production of proinflammatory and antiviral mediators in response to Poly(I:C) [80]. Moreover, compared to AMs of never-smokers, poly(I:C)-stimulated production of CXCL10 was significantly reduced in AMs of smokers [79].

Our observation of the impairment of TLR3-mediated M2 to M1 phenotype shift mediated by CSE is in accordance with above-cited studies, and this effect of CSE represents an additional mechanism to explain the prevalence of M2 cells often observed in lung microenvironment of smokers. Accordingly, the lung is constantly exposed to exogenous TLRs ligands, including chemicals, dust, pollen and especially microorganisms expressing unique microbial patterns called Pathogen associated molecular patterns (PAMPs) [81, 82] such as Lipopolysaccharide (LPS) in outer membrane of gram-negative bacteria, lipoteichoic acid and peptidoglycan in cell wall of gram-positive bacteria, flagellin of bacterial flagella, dsRNA and ssRNA of viruses etc. [83–85] selectively recognized by the TLRs. Thus the inhibition of the TLR3-mediated M2 macrophages phenotype shift towards the M1 could increase M2 cells fraction and consequently reduce the immune activation against the tumor.

Here we also showed that M2-phenotype macrophages have a lower sensibility to CSE compared to M0 cells. The reason why M2-phenotype cells appear to be more resistant than naïve macrophages is still under investigation. High resistance of M2-phenotype macrophages to CSE cytotoxic activity could represent another additional cause to explain the high frequency of M2 cells in the lung microenvironment of smokers.

CSE reduces the capability of both cancer cells and macrophages to proliferate *in vitro*, and the extent of proliferation reduction is proportional to CSE concentration and time of exposure. Macrophages were more sensitive to CSE exposure than cancer cells. Moreover, as regards the macrophages interaction with tumor cells, our results indicate that cancer cells are able to drive CSE resistance in co-cultured RAW cells. This observation could be due to the CSE-induced shift of RAW cells towards a more CSE-resistant phenotype such as M2, able to support its own growth.

## Conclusions

Obtained results indicate that CS affects phenotype and viability of macrophages. CS induces macrophages polarization towards the M2 phenotype and M2-phenotype macrophages resulted less sensitive to the CS cytotoxic activity, compared to M0 and M1 phenotype cells. Our results also suggest that an additional mechanism for M2 enrichment in lung smokers could be the impairment of the TLR3-mediated M2-M1 macrophage phenotype shift by CS. In conclusion, these findings indicate that, in lung cancer microenvironment of smokers, CS can contribute to the M2-phenotype macrophages prevalence by different mechanisms, which can be in part challenged. Ultimately, the increase of the anti-inflammatory macrophage population could explain the worse prognosis of smokers compared to non-smokers.

## Supporting information

S1 File (PDF)

## List of abbreviations

NSCLC: Non-Small Cell Lung Cancer
AMs: Alveolar macrophages
TAMs: tumor-associated macrophages
CS: cigarette smoke
2D: two-dimensional
3D: three-dimensional
CSE: cigarette smoke extract
FBS: fetal bovine serum
SRB: Sulforhodamine B
OD: optical density
CFSE: carboxyfluorescein succinimidyl ester
TLR: Toll-like receptor
