# Supplementary material for: Cigarette smoke sustains immunosuppressive microenvironment inducing M2 macrophage polarization and viability in lung cancer settings
Source: PLoS One. 2024 May 22;19(5):e0303875. doi: 10.1371/journal.pone.0303875 (PMC11111031; doi:10.1371/journal.pone.0303875)
Supplement: S1 File — (PDF) [file pone.0303875.s001.pdf]

Minimal data set to consist of the data required to replicate all study findings reported in the article

Figure 1

- The values behind the means, standard deviations and other measures reported;

- The values used to build graphs.

– Panel B

RAW	RAW+IFN-γ	RAW+LLCsur
0,0254728	0,06077913	0,03410717
0,02458732	0,1112081	0,01018521
0,02034131	0,1606548	0,01035044
0,025640	0,094740	0,011290

RAW	RAW+IFN-γ	RAW+LLCsur
0,01980473	0,009335829	0,03126448
0,01708502	0,00544679	0,006008679
0,008748688	0,004252627	0,008916133

– Panel C

RAW	RAW+IFN-γ	RAW+LLCsur
0,01669218	0,01502938	0,01149777
0,0125859	0,03749924	0,01866937
0,01216165	0,03644423	0,0192235
0,01837999	0,04456175	0,02336948

– Panel D

RAW	RAW+IFN-γ	RAW+LLCsur
0,01980473	0,009335829	0,03126448
0,01708502	0,00544679	0,006008679
0,008748688	0,004252627	0,008916133

– Panel E

RAW	RAW+IFN-γ	RAW+LLCsur
0,001360	0,000110	0,001000
0,001280132	0,000373568	0,007146017
0,000908056	0,000283797	0,006073832

– Panel F

RAW	RAW+IFN-γ	RAW+LLCsur
0,0000161993	0,000334264	0,000184279
0,000012816	0,000303087	0,000295666
0,00000996837		0,000259239

– Panel G

RAW	RAW+IFN-γ	RAW+LLCsur
0,0000113196	0,000519091	0,007264814
0,0000103704	0,000422784	0,007018715
0,00000943357	0,000389808	0,006899107

Figure 2

- The values behind the means, standard deviations and other measures reported;

- The values used to build graphs.

– Panel B

M1	M1 + CSE	M0	M0+CSE
0,023300	0,018500		
0,020355	0,022604		
0,013069	0,013270		
		0,01520375	0,01624353
		0,01477979	0,01765994

Number of values	3	3	2	2
Minimum	0,01307	0,01327	0,01478	0,01624
25% Percentile	0,01307	0,01327	0,01478	0,01624
Median	0,02036	0,0185	0,01499	0,01695
75% Percentile	0,0233	0,02260	0,01520	0,01766
Maximum	0,0233	0,02260	0,01520	0,01766
Mean	0,01891	0,01812	0,01499	0,01695
Std. Deviation	0,005267	0,004678	0,0002998	0,001002
Std. Error	0,003041	0,002701	0,0002120	0,0007082
Lower 95% CI of mean	0,005825	0,006503	0,01230	0,007953
Upper 95% CI of mean	0,03199	0,02975	0,01769	0,02595

– Panel C

M1	M1 + CSE	M0	M0+CSE
0,051400	0,055100		
0,024326	0,027057		
0,014851	0,017840		
		0,03923397	0,06422149
		0,04232435	0,07591812

	M1	M1 + CSE	M0	M0+CSE
Number of values	3	3	2	2
Minimum	0,01485	0,01784	0,03923	0,06422
25% Percentile	0,01485	0,01784	0,03923	0,06422
Median	0,02433	0,02706	0,04078	0,07007
75% Percentile	0,0514	0,0551	0,04232	0,07592
Maximum	0,0514	0,0551	0,04232	0,07592
Mean	0,03019	0,03333	0,04078	0,07007
Std. Deviation	0,01897	0,01941	0,002185	0,008271
Std. Error	0,01095	0,01120	0,001545	0,005848
Lower 95% CI of mean	-0,01693	-0,01488	0,02115	-0,004240
Upper 95% CI of mean	0,07731	0,08154	0,06041	0,1444

Figure 2 – Panel D

M1	M1 + CSE	M0	M0+CSE
0,001162	0,001131		
0,2633717	0,1295524		
		0,0000571703	0,00010338
		0,0000669536	0,000108433

	M1	M1 + CSE	M0	M0+CSE
Number of values	2	2	2	2
Minimum	0,001162	0,001131	5,717e-005	0,0001034
25% Percentile	0,001162	0,001131	5,717e-005	0,0001034
Median	0,1323	0,06534	6,206e-005	0,0001059
75% Percentile	0,2634	0,1296	6,695e-005	0,0001084
Maximum	0,2634	0,1296	6,695e-005	0,0001084
Mean	0,1323	0,06534	6,206e-005	0,0001059
Std. Deviation	0,1854	0,09081	6,918e-006	3,573e-006
Std. Error	0,1311	0,06421	4,892e-006	2,527e-006
Lower 95% CI of mean	-1,534	-0,7505	-9,228e-008	7,380e-005
Upper 95% CI of mean	1,798	0,8812	0,0001242	0,0001380

Figure 3

– Panel A

M2	M2 + PolyIC	M2 + PolyIC + CSE 5%
0,01505703	0,03428629	0,01368205
0,01714233	0,02678411	0,01429033

– Panel B

M2	M2 + PolyIC	M2 + PolyIC + CSE 5%
0,000368	0,003748	0,002213
0,000304	0,003443	0,002220

– Panel C and D

- The values used to build graphs and the values behind the means, standard deviations and other measures reported

M2	M2+Poly(I:C)	M2+Poly(I:C)+CSE
5,09	60,1	45,1
9,35	70,6	46,3

M2	M2+Poly(I:C)	M2+Poly(I:C)+CSE
0,00	8,52	3,22
0,57	10,00	2,78

Figure 4

– Panel A

- The values used to build graphs and the values behind the means, standard deviations and other measures reported

time	CSE	average ratio	OD						ratio					
24	NT	1	0,2385	0,2388	1,1295			0,3582	1	1	1			1
	5%	0,887340265	0,178	0,2608				0,295	0,746331	1,092127				0,823562
	10%	0,62562099	0,1655	0,2208	0,28325			0,2268	0,69392	0,924623	0,250775			0,633166
72	NT	1	0,8965	1,67525	1,94575	1,023	2,926833	1,4264	1	1	1	1	1	1
	5%	0,680940589	0,443	1,1154		0,6746	1,742667	1,412	0,494144	0,665811		0,659433	0,59541	0,989905
	10%	0,486543385	0,2575	0,6548	0,589			1,377	0,287228	0,390867	0,302711			0,965367

– Panel B

- The values used to build graphs

ratio

M0	M0+CSE	M2	M2+CSE
1,000000	0,3157895	1,00	0,9879519
1,000000	0,680000	1,00	0,7272727
1,000000	0,740000	1,00	0,730000
1,000000	0,560000	1,00	1,160000
		1,00	0,840000

- The values behind the means, standard deviations and other measures reported

Raw data

M0 NT	M0+CSE	M2 NT	M2+CSE
1900000,00	600000,00	2766666,00	2733333,00
2900000,00	1966666,00	4033333,00	2933333,00
3200000,00	2366666,00	4200000,00	3066666,00
5966000,00	3312000,00	3900000,00	4533332,00
		7200000,00	6066000,00

– Panel C

- The values used to build graphs

ratio

M0	M0+CSE	M2	M2+CSE
1,	0,670000	1,	0,8492997
1,	0,710000	1,	0,9007708
1,	0,600000	1,	0,9695536
1,	0,660000		
1,	0,8154056		

- The values behind the means, standard deviations and other measures reported

Raw data

M0	M0+CSE 5%
1,675250	1,115400
0,896500	0,443000
2,926833	1,742667
1,530000	1,010000
2,079333	1,695500
M2	M2+CSE 5%
1,071000	0,909600
1,881167	1,694500
1,691500	1,640000

– Panel D

- The values used to build graphs

ratio

M0	M0+CSE	M2	M2+CSE
1,0	0,6538461	1,00	0,8333333
1,0	0,720000	1,00	1,153846
1,0	0,5973154	1,00	0,6244898
1,0	0,6237624		

- The values behind the means, standard deviations and other measures reported

Raw data

M0	M0+CSE	M2	M2+CSE
26,0	17,0	15,0	12,5
25,0	18,0	13,0	15,0
14,9	8,9	8,2	5,1
13,5	8,4		

Figure 5

- The values used to build graphs

ratio

RAW	RAW+CSE	RAW/LLC	RAW/LLC+CSE
1,0	0,797048	1,0	0,685714
1,0	0,797048	1,0	0,900000
1,0	0,9446495	1,0	0,728571
1,0	1,003690	1,0	1,071429
1,0	0,6199262	1,0	0,985714
1,0	0,6199262		
1,0	0,501845		

- The values behind the means, standard deviations and other measures reported

Raw data

RAW	RAW+CSE	RAW/LLC	RAW/LLC+CSE
3,7	2,7	2,5	1,6
3,5	2,7	3,1	2,1
2,8	3,2	2,7	1,7
3,3	3,4	2,4	2,5
2,8	2,1	1,8	2,3
3,2	2,1	1,5	
3,9	1,7		

statistics	RAW	RAW+CSE	RAW/LLC	RAW/LLC+CSE
Number of values	7	7	6	5
Minimum	2,800	1,700	1,500	1,600
25% Percentile	2,900	2,100	1,725	1,650
Median	3,400	2,700	2,450	2,100
75% Percentile	3,850	3,200	2,800	2,400
Maximum	3,900	3,400	3,100	2,500

Mean	3,388	2,557	2,333	2,040
Std. Deviation	0,4422	0,6214	0,5888	0,3847
Std. Error	0,1563	0,2349	0,2404	0,1720
Lower 95% CI of mean	3,018	1,982	1,715	1,562
Upper 95% CI of mean	3,757	3,132	2,951	2,518

Figure 6

– Panel B and C

- The values used to build graphs

ratio	M1/LLC1	M1/LLC1+CSE	M2/LLC1	M2/LLC1+CSE
ratio 3	1,000000	1,020000	1,000000	1,410000
ratio 3b	1,000000	0,800000	1,000000	1,890000
ratio 3c	1,000000	1,010000	1,000000	1,140000

ratio	M1/LLC1	M2/LLC1	M1/LLC1	M2/LLC1
ratio 3	1,000000	1,290000	1,000000	1,790000
ratio 3b	1,000000	0,990000	1,000000	2,360000
ratio 3c	1,000000	1,290000	1,000000	1,480000

- The values behind the means, standard deviations and other measures reported

raw data	M1/LLC1+CSE	M2/LLC1+CSE
exp. 3		
M1+LLC+CSE120.tif	7,750000	
M1+LLC+CSE121.tif	5,160000	
M1+LLC+CSE122.tif	6,730000	
M1+LLC+CSE123.tif	6,260000	
M2+LLC1+CSE31.tif		10,800000
M2+LLC1+CSE32.tif		11,290000
M2+LLC1+CSE33.tif		8,120000
M2+LLC1+CSE34.tif		16,110000
exp. 3b		
M2+CSE+LLC152.nd2		21,930000
M2+CSE+LLC150.nd2		31,750000
M2+CSE+LLC148.nd2		41,440000
M2+CSE+LLC146.nd2		40,370000
M2+CSE+LLC145.nd2		37,410000
M1+LLC1+CSE24.nd2	13,610000	
M1+LLC1+CSE22.nd2	13,070000	
M1+LLC1+CSE20.nd2	13,820000	
M1+LLC1+CSE18.nd2	18,060000	
exp.3c		
8	5,800000	
1	18,740000	
7	10,140000	
4		19,820000
5		19,420000
10		28,070000

raw data	M2/LLC1	M2/LLC1+CSE
exp.3		

M2+LLC124.tif	5,210000	
M2+LLC125.tif	6,110000	
M2+LLC126.tif	6,940000	
M2+LLC127.tif	8,600000	
M2+LLC128.tif	10,260000	
M2+LLC129.tif	13,780000	
M2+LLC130.tif	6,500000	
M2+LLC1+CSE31.tif		10,800000
M2+LLC1+CSE32.tif		11,290000
M2+LLC1+CSE33.tif		8,120000
M2+LLC1+CSE34.tif		16,110000
exp. 3b		
M2+CSE+LLC152.nd2		21,930000
M2+CSE+LLC150.nd2		31,750000
M2+CSE+LLC148.nd2		41,440000
M2+CSE+LLC146.nd2		40,370000
M2+CSE+LLC145.nd2		37,410000
M2+LLC138.nd2	15,450000	
M2+LLC136.nd2	17,010000	
M2+LLC134.nd2	17,290000	
M2+LLC132.nd2	21,070000	
M2+LLC131.nd2	20,650000	
exp. 3c		
3	10,310000	
9	12,770000	
6	17,020000	
4		19,820000
5		19,420000
10		28,070000

statistics	M2/LLC1	M2/LLC1+CSE
Number of values	15	12
Minimum	5,210	8,120
25% Percentile	6,940	12,50
Median	12,77	20,88
75% Percentile	17,02	36,00
Maximum	21,07	41,44
Mean	12,60	23,88
Std. Deviation	5,344	11,76
Std. Error	1,380	3,395
Lower 95% CI of mean	9,639	16,41
Upper 95% CI of mean	15,56	31,35

	M1/LLC1+CSE	M2/LLC1+CSE
Number of values	11	12
Minimum	5,160	8,120
25% Percentile	6,260	12,50
Median	10,14	20,88
75% Percentile	13,82	36,00
Maximum	18,74	41,44
Mean	10,83	23,88
Std. Deviation	4,916	11,76

Std. Error	1,482	3,395
Lower 95% CI of mean	7,529	16,41
Upper 95% CI of mean	14,13	31,35

Supplementary Figure 3

– Panel A

M0	M0+CSE	M1	M1+CSE
8,21	16,8	35,5	44,8
20,80	37,1	23,1	7,4

– Panel B

M0	M0+CSE	M1	M1+CSE
6,19	13,8	22,8	14,
11,00	13,8	61,8	71,
17,10	19,6		
